# A comparative study of ripening among berries of the grape cluster reveals an altered transcriptional programme and enhanced ripening rate in delayed berries

**DOI:** 10.1093/jxb/eru329

**Published:** 2014-08-18

**Authors:** Satyanarayana Gouthu, Shawn T. O’Neil, Yanming Di, Mitra Ansarolia, Molly Megraw, Laurent G. Deluc

**Affiliations:** ^1^Oregon Wine Research Institute, Oregon State University, Corvallis, OR 97331, USA; ^2^Department of Horticulture, College of Agricultural Sciences, Oregon State University, Corvallis, OR 97331, USA; ^3^Center For Genome Research and Biocomputing, Oregon State University, Corvallis, OR 97331, USA; ^4^Department of Statistics, College of Sciences, Oregon State University, Corvallis, OR 97331, USA; ^5^Department of Botany and Plant Pathology, College of Agricultural Sciences, Oregon State University, Corvallis, OR 97331, USA

**Keywords:** Enhanced ripening, grape, hormone, plasticity, ripening synchronization, transcriptional programme.

## Abstract

Transcriptional studies in relation to fruit ripening generally aim to identify the transcriptional states associated with physiological ripening stages and the transcriptional changes between stages within the ripening programme. In non-climacteric fruits such as grape, all ripening-related genes involved in this programme have not been identified, mainly due to the lack of mutants for comparative transcriptomic studies. A feature in grape cluster ripening (*Vitis vinifera* cv. Pinot noir), where all berries do not initiate the ripening at the same time, was exploited to study their shifted ripening programmes in parallel. Berries that showed marked ripening state differences in a véraison-stage cluster (ripening onset) ultimately reached similar ripeness states toward maturity, indicating the flexibility of the ripening programme. The expression variance between these véraison-stage berry classes, where 11% of the genes were found to be differentially expressed, was reduced significantly toward maturity, resulting in the synchronization of their transcriptional states. Defined quantitative expression changes (transcriptional distances) not only existed between the véraison transitional stages, but also between the véraison to maturity stages, regardless of the berry class. It was observed that lagging berries complete their transcriptional programme in a shorter time through altered gene expressions and ripening-related hormone dynamics, and enhance the rate of physiological ripening progression. Finally, the reduction in expression variance of genes can identify new genes directly associated with ripening and also assess the relevance of gene activity to the phase of the ripening programme.

## Summary

The developmental programme of grape berries within a cluster is coordinated to synchronize their ripening. Altered transcriptional events and metabolite accumulation are responsible for the differential progress of ripening.

## Introduction

Ripening in fleshy fruits is a highly coordinated and genetically programmed process involving changes in specific biochemical and physiological attributes. During this developmental programme, genes with a wide range of biological functions are recruited and their expression is coordinately modulated in a phase-specific manner (Klee and Giovannoni, 2011; Gapper *et al.*, 2013; Osorio *et al.*, 2013).

In grapevine, the programme of fruit ripening lasts for up to ~60 d from véraison (onset of ripening) until maturity, and the berries of a cluster are generally timed to go through the programme simultaneously. The biochemical and physiological stages and associated transcriptional states have been reported, which help in understanding the overall transcriptional programme of ripening (Deluc *et al.*, 2007; Lund *et al.*, 2008; Zenoni *et al.*, 2010; Ali *et al.*, 2011). However, berries in a véraison cluster enter the ripening programme asynchronously, resulting in the appearance of different ripening phenotypes that are developmentally up to 2 weeks apart in terms of sugar and pigment levels. During the later stages of development towards maturity, these ripening-related differences will reduce and synchronize among the berries. Based on separate studies, the extent of the metabolic synchronization seems to depend on the genotype, environmental conditions, and internal hormone dynamics (Böttcher *et al.*, 2010a; Pagay and Cheng, 2010; Dai *et al.*, 2011). These observations indicate that the ripening programme is flexible and some of its aspects, including the rate of ripening progression, can be modulated depending upon environmental factors and developmental demands. In rice, which also has panicle inflorescence structure, modulated ripening programmes including in gene expression, enzyme activity, and accumulation of soluble sugars are observed between the grains situated at distal and proximal positions of the inflorescence (Ishimaru *et al.*, 2005). Usually altered growth responses to external and internal cues (Walch-Liu *et al.*, 2000; Bonhomme *et al.*, 2012) are possible when genes responsive to the stimulus deviate from their normal developmental expression patterns (Swindell *et al.*, 2007).

Changes in the cellular concentrations of important signalling molecules such as sugars affect the ripening process by influencing the expression of large networks of genes (Rolland *et al.*, 2006; Matsoukas *et al.*, 2013). Unlike in climacteric fruits, ethylene is not considered a major regulator in non-climacteric fruits, other than a few recent reports of its slight increase and expression of auxin (indole acetic acid; IAA) biosynthetic genes in response to treatment with the ethylene-releasing compound, Ethrel (Chervin *et al.*, 2004; Böttcher *et al.*, 2013). Incidentally, IAA is regarded as an inhibitor of fruit ripening, and abscisic acid (ABA) as a ripening promoter during grape berry ripening (Davies and Böttcher, 2009; Böttcher *et al.*, 2010a).

In the developmental context, studies in grape have focused on the differences between different stages of cluster ripening, with the underlying concept that all berries have the same duration of ripening programme, irrespective of their differences in ripening initiation times (Deluc *et al.*, 2007; Zenoni *et al.*, 2010; Lijavetzky *et al.*, 2012). At the same time, the extent of expression differences between the stages was used as the main criterion to assess the relevance of genes in the ripening programme. Other studies reported transcriptional alterations in the ripening programme due to external factors (water stress, light, temperature) (Deluc *et al.*, 2009; Carbonell-Bejerano *et al.*, 2013; DalSanto *et al*., 2013). However, no attempt was made to compare the ripening programmes between the berries of the same cluster where the duration of the programmes from véraison to maturity are seemingly different. In the present study, the physiological and transcriptional states were examined in three different ripening classes of berries at their véraison and maturity stages separately, and it was shown that they synchronize between berry classes at maturity. Along with the progression of physiological ripening, the model that emerged from the present study demonstrates that the transcriptional programme of ripening is the same among the different classes of berries, but its duration is flexible. Additionally, by examining the genes that are required to alter their expression dynamics most, during the synchronization of ripening, it is shown that genes associated with ripening can be identified more accurately.

## Materials and methods

### Plant material and sampling of berry ripening-phenotype classes

**Fig. 1.**
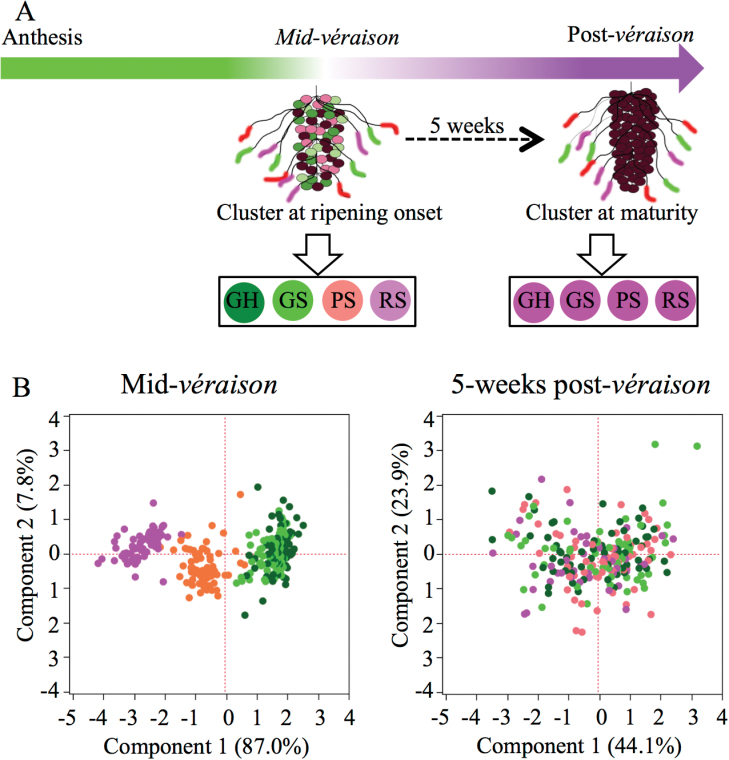
Ripening state variation among berries within a cluster at ripening onset and uniform ripeness upon maturity. (A) Schematic representation of cluster ripening and sampling of ripening classes Green Hard (GH), Green Soft (GS), Pink Soft (PS), and Red Soft (RS) at mid-véraison (V) and at 5 weeks post-véraison (PostV). (B) Principal component analyses showing ripening differences between GH, GS, PS, and RS berries at V and PostV. Colour [L (lightness), h (hue angle), and C (chroma)], and total soluble solid content (°Brix) of individual berries were used as continuous variables. The number of berries (*n*) is 75 for each class in the V plot; and 67, 61, 64, and 47 berries for GH, GS, PS, and RS, respectively, for the PostV plot. Dots representing GH, GS,PS or RS berries are clustered from right to left, respectively, in the mid-véraison plot. Loading plot data of the PCAs are given in Supplementary Table S6 at *JXB* online. (This figure is available in colour at *JXB* online.)

Experiments were conducted at the Oregon State University research vineyard in Alpine, Oregon during 2010 and 2011. *Vitis vinifera* L. cv. Pinot noir clone Pommard grafted to 101–14 rootstock used for these experiments was trained in a double guyot system with vertically positioned shoots. Five primary clusters each were selected from three vines located in different rows from either side of the vine canopy. Sixty-nine days after anthesis, when 50% of the berries in the cluster had changed colour (mid-véraison; V), the ripening classes Green Hard (GH), Green Soft (GS), Pink Soft (PS), and Red Soft (RS) were identified by touch and visual colour perception. From each cluster, five berries belonging to each class were sampled at V and five berries, still on the clusters, were tagged with colour-coded strings to identify the initial ripening class (Fig. 1). The tagged berries were then sampled 5 weeks after mid-véraison (PostV). Analysis of variance (ANOVA) was used to test for differences in total soluble solids (TSS: °Brix) and colour content between clusters and plants. The ripening states of berries were determined using TSS, colour, and elasticity (MPa) measurements. Berry elasticity was measured using customized springs in a Harpenden skinfold caliper (Baty International, UK) (Thomas *et al.*, 2008). TSS was measured using a digital refractometer (SPER Scientific Inc., USA). Berry colour parameters, namely L (lightness), h (hue angle), and C (chroma), were measured using a Minolta C-300 colorimeter (Minolta Corp, Osaka, Japan) at the berry equator. Colour index was calculated as 180h/L+C (Carreno and Martinez, 1995). In 2011, to assess the ripening progress in each berry class, the experiment was conducted on five clusters each of six plants, and one berry per ripening class was collected from each cluster at weekly intervals following V (at 0, 1, 2, 3, 5, and 6 weeks).

### Berry selection for transcriptional studies

Discriminant analysis of the berries (JMP-9; SAS USA) was performed on the parameters of TSS, colour, and elasticity of berries from each plant. Ripening class served as a discriminant parameter, and berries that most represent a ripening class were selected. Skin, pulp, and seed tissues from selected berries from each plant were pooled separately for RNA extraction.

### Transcriptomics and data analysis

Details of RNA isolation, cDNA synthesis, hybridization with NimbleGen *Vitis vinifera* Gene Chips, and data processing are presented in the Supplementary text available at *JXB* online. The expression data are available at Gene Expression Omnibus (serial number: GSE49569) (http://www.ncbi.nlm.nih.gov/geo/). Moderated paired *t*-tests were used to compare the mean log expression intensities between berry classes (GS compared with PS, PS compared with RS, and GS compared with RS) at V and PostV [R/Bioconductor Limma package (Smyth, 2004; R Development Core Team, 2012)] with the false discovery rate at FDR <0.05 (Benjamini and Hochberg, 1995). Genes that passed the significance threshold between any of the two berry classes were considered differentially expressed, and these gene sets were used for the following assessments. Normalization of the transcriptomic data is described in the Supplementary text.

### Calculation of reduction in variance (RV) in gene expression

Variance between the expression values of berry classes were calculated independently at V and PostV. The ratio of these two variances is expressed as fold RV. The significance of RV was tested using an *F*-test for expression variances (FDR <0.2).

### Gene expression trends from V to PostV

Patterns of the expression progress from V to PostV (up-regulated, down-regulated, or plateaued) were determined by comparing the mean expression level among berry classes at PostV with that of véraison RS berries using a two-sided contrast analysis (FDR <0.05) (Bretz *et al.*, 2010). Genes with significant changes in expression were designated as up- or down-regulated depending on their expression level at PostV relative to that at véraison RS. Genes with no significant change in expression were considered to have reached a plateau and maintained the expression from V to PostV. These data were used to examine the correlation between the trends in gene expression and the variance of gene expression among the berry classes.

### Calculation of transcriptional distances

Expression differences between berry classes were determined separately at V and at PostV stages, which are representative transcriptional distances between transitional stages. To assess V to PostV expression changes in each berry class, the expression difference of a given berry class from the normalized V and PostV data was calculated. These data represent the transcriptional distance for a gene from the véraison ripening stage to that of the PostV ripening phase in each of the berry class. Differences in GS–PS and PS–RS at V are indicated as **a or b**, respectively, in the transcriptional distance model (Fig. 3). The changes in expression from V to PostV stages between GS, PS, and RS berry classes are represented as X, Y, and Z respectively.

### Calculation of ripening rates

Regression equations were obtained from the ripening progress models based on TSS (°Brix) and colour index in each berry class over a 2 week period. The starting points of the curves were determined as the point at which all classes (GH, GS, and PS) had reached TSS and colour index levels characteristic of RS berries at V. The number of days for under-ripe berry classes to reach the TSS and colour levels of RS berries at 0, 7, and 14 d (R1, R2, and R3 stages) was estimated using the regression equations derived from each class. These data were used to calculate ripening rates (Supplementary text at *JXB* online).

### Identification of ripening equivalent stages

The TSS and colour index values, recorded in RS berries at V and at 1, 2, and 3 weeks past V, were used to define the reference ripening states (R1, R2, R3, and R4). Ripening equivalent stages were defined as the stages at which GS berries had reached similar TSS and colour index values to those of the RS reference berries.

### Plant hormone analysis

Pulp tissue of GS and RS berries at the ripening equivalent stages of R1, R2, and R3 were used for the analysis of ABA, IAA, and gibberellin 4 (GA_4_) concentrations. Purified hormones from 50mg of tissue were quantified by liquid chromatography–tandem mass spectrometry analysis using a hybrid triple quadrupole/linear ion trap 4000 QTRAP LC-MS/MS instrument in the MRM mode (Gouthu *et al.*, 2012).

### Quantitative real-time RT–PCR analysis

Gene expression levels in berry pulp tissue were analysed with the SYBR Green assay using an ABI 7500 Fast Real-Time PCR System (Applied Biosystems). The cDNAs prepared from RNA samples from three biological and two technical replicates were used in the PCRs, and the peptidyl-prolyl *cis-trans* isomerase gene (VIT_06s0004g06610) was used for data normalization. The ΔΔC_t_ method was followed for quantification of expression levels (Supplementary text at *JXB* online).

### Identification of transcriptional modules during the ripening transitions

The data integration framework ‘DISTILLER’ was used to identify transcriptional modules (co-expressed genes sharing over-represented *cis*-regulatory elements or motifs) in the data (Lemmens *et al.*, 2009). Over-represented motifs in the data set and position weight matrix data were extracted using the DREME algorithm (Bailey, 2011), and motif scores of zero or one were assigned to individual genes depending on the significant presence of the motif site (see Supplementary text at *JXB* online). This binary matrix is used as input data for DISTILLER to identify transcriptional modules.

### Gene ontology (GO) enrichment analysis

Enrichment analyses were performed using the AGRIGO online tool (Du *et al.*, 2010). Over-represented Plant GO slim categories were identified using a single enrichment analysis and applying a hypergeometric test with the FDR set at <0.1.

## Results

### Ripening differences among berries of the cluster at véraison and post-véraison

Grape clusters are at véraison ~60 d post-anthesis [at the E-L 35 phenological stage (Coombe, 1995)] depending on the cultivar. Because berries enter the ripening phase at different times, green berries that are hard (GH) or soft (GS), pink soft berries (PS), and red soft berries (RS) exist together in a cluster at mid-véraison (V), but the visual differences between berries disappear once green berries develop colour (Fig. 1A). The berry classes sampled at V, and the same classes that were tracked and sampled at 5 weeks post-véraison (PostV; E-L 38), were assessed for ripening status based on objective sugar content measured as TSS, and pigment level measured as colour parameters (L, C, and h). Sugar and colour were found to be reliable discriminant parameters until maturity in the present data, in agreement with other studies, and even subtle changes in colour at PostV could be detected (Kontoudakis *et al.*, 2011). Berry classes were distinct from each other at V in a principal component analysis (PCA) performed using the ripening features of individual berries (Fig. 1B; left panel). No or few differences were found between berries of a class collected from different clusters or different experimental plants (Supplementary Table S1 at *JXB* online). PCA of berries did not show separation of classes at PostV (Fig. 1B; right panel), and ANOVA between classes showed no significant differences (*P*>0.05). Based upon probability scores from discriminant analyses, 70–99% of berries within each class were similar to their respective classes at V (Supplementary Table S2). At PostV, berries within each class exhibited a greater degree of variation that caused each class to lose its unique ripeness identity.

### Transcriptional differences among the berries of the cluster at véraison and post-véraison

To examine whether transcriptional differences between the ripening classes of a cluster at V persist until maturity, expression profiling, using genome-wide arrays, was performed at the physiological maturity stage of 5-week post-véraison rather than an intermediate developmental point.

**Fig. 2.**
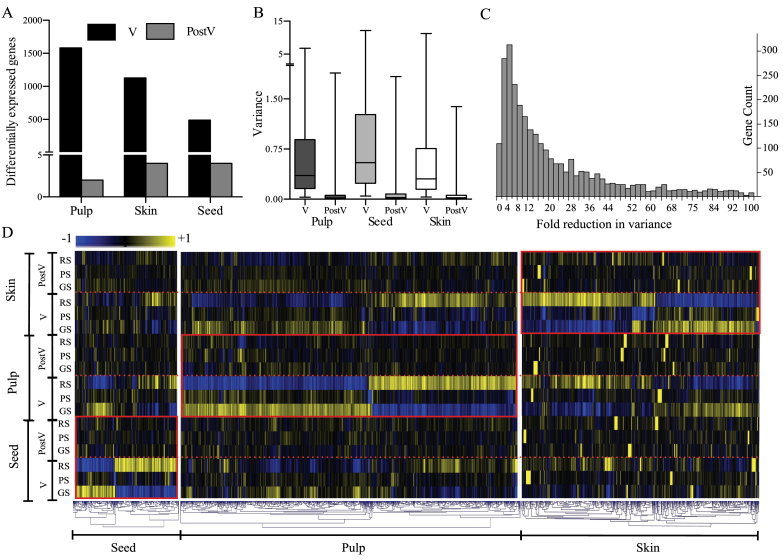
Reduction of transcriptional variance between GS, PS, and RS berry ripening classes from mid-véraison (V) to 5 weeks post-véraison (PostV). (A) Numbers of genes differentially expressed between berry classes in pulp, skin, and seed at V and PostV (paired *t*-tests, FDR <0.05). (B) Ranges of transcriptional variances (variance of expression levels between GS, PS, and RS) among the differentially expressed genes at V and the corresponding variances at PostV in pulp, skin, and seed (variances at PostV were non-significant, FDR >0.05). (C) Distribution of genes that showed 2- to 100-fold reduction in transcriptional variance (RV) from V to PostV. Eighty-five percent of the synchronizing genes fall within the 100-fold reduction range. (D) Hierarchical clustering analysis of the genes differentially expressed between the three berry classes at V. TMev 4.3 software was used to draw the dendrogram with Pearson’s correlation distance as the metric. Rectangular boxes enclose expression maps of differentially expressed genes at V and of the same genes at PostV in each tissue. Dotted lines separate V and PostV data sets. Differences in expression levels between GS, PS, and RS for these genes were not significant at PostV. Expression maps of the corresponding gene sets from other tissues, regardless of differential expression, are shown to indicate similarities in their expression patterns. (This figure is available in colour at *JXB* online.)

Because softening of the berry is the first clear indication of the beginning of the ripening process, GS was considered the first ripening stage in the present transcriptomic study. In the PCA of the expression data of all 54 samples, the first and second principal components together explained 51.4% of the variability (Supplementary Fig. S1 at *JXB* online). The results of PCA showed consistency across biological replicates, tissues, and ripening stage times, with some variability in pulp at V. At V, 1583, 1126, and 491 differentially expressed genes were identified between the three berry classes (GS, PS, and RS) that play a role in véraison transitions in pulp, skin, and seed, respectively (FDR <0.05) (Fig. 2A; Supplementary Worksheet S1A). The majority of the differentially expressed genes had at least a 2-fold expression difference between any of the two berry classes (Supplementary Worksheet S1A). Among these, none of the genes showed differential expression at PostV, and their expression variances were reduced to various extents, resulting in synchronized transcriptional states among the berry classes. The range of reduced expression differences among berry classes was apparent from the variance ranges in V and PostV data sets (Fig 2B; Supplementary Worksheet S1B). Only a few genes, two from pulp, four from seed, and four from skin, which were not among those differentially expressed at V, showed significant expression differences at PostV (Supplementary Worksheet S1C). Variability associated with replicates was not a major contributing factor to the expression variance values as the coefficient of variation between replicates for >70% of the genes was ≤10% (Supplementary Worksheet S1D).

### Reduction in expression variance between berry classes

The sample distribution of the reduction in variance (RV), expressed as the ratio between gene expression variances at V and PostV, was skewed with the mean at 24-fold change (Fig. 2C). *F*-tests performed to compare V and PostV variances show significant RV (FDR <0.2) in >80% of the genes (Supplementary Worksheet S1B at *JXB* online). Neither the level of expression variance among the berry classes nor the pattern of expression at V correlated to the extent of RV in any of the tissues (Fig. 2D). Hierarchical clustering of genes differentially expressed in one tissue compared with the expression trends of the corresponding genes in the other two tissues indicated more commonality in the expression behaviour between skin and pulp tissues (Fig. 2D). In general, the level of expression of a gene seemed to impact its expression variance at both V and PostV. The distribution of expression variance among the low and highly expressed genes showed that the majority of the genes in the low expression set had several fold higher variance compared with those in the high expression set, and the same trend extended to PostV expression variances (Supplementary Fig. S2). The relationship between the trends of expression from véraison to maturity and RV was further examined. Similar proportions of genes showed up-regulated, down-regulated, or plateaued expression trends from véraison RS to PostV stages (Supplementary Worksheet S1B). Up-regulated expression was predominant among genes exhibiting the most RV. Twice as many genes were up-regulated from V to PostV within the set of high RV genes (>20-fold RV) compared with the set of low RV genes (<10-fold RV). In contrast, twice as many genes were down-regulated in the low RV set relative to the high RV set (Supplementary Table S3). Because the experiment was primarily aimed at the assessment of the véraison expression variances among berry classes and their fate at maturity stage, the phase-specific extent of RV during the course of ripening could not be identified. However, the V to PostV expression trends gave an indication of the timing of maximum RV at early or late stages. In contrast to up- or down-regulated trends, where a gradual reduction in variance is visualized, plateaued expression indicates that maximum RV occurs before PostV. GS and PS berries reach their respective RS states within 2 weeks past véraison, as shown in the later sections; as their transcriptional states at PostV were the same as those of the original RS berries and these genes undergo no expression change after the RS stage, the timing of synchronization was considered here as the early post-véraison period. Single enrichment analysis for enriched GO terms (Du *et al.*, 2010) in this gene set showed enrichment of several genes in primary and secondary metabolism that would be expected to have the highest functional relevance during the early ripening changes (Supplementary worksheet S1E).

### Assessment of ripening-associated genes using reduction of variance score

High-throughput expression studies are often used to hypothesize a relationship between differential expression and the importance of genes to a biological process. Here the data were examined based on both expression variance between the ripening stages and the scores of RV between berry classes in order to compare the two assessment methods in finding the genes associated with ripening. Among the sets of top 100 genes identified from the two methods, only 15% of genes were common and could be considered highly relevant to ripening (Supplementary Table S4 at *JXB* online). Using RV as the criterion, most of the genes would have been considered as low priority if they had only been ranked based on their expression variance. Since the RV score additionally considers the requirement for genes to reduce their expression differences between green and riper berries, which describes the synchronization of their ripening states toward maturity, these genes identified are more relevant to the ripening.

### Defined expression changes (transcriptional distances) between ripening stages

**Fig. 3.**
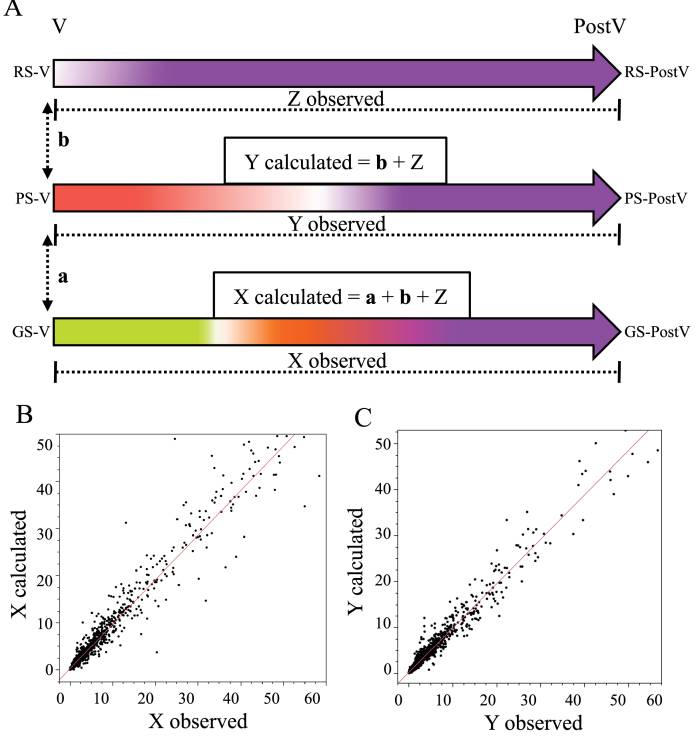
Transcriptional programme of ripening derived from the expression data of berry classes at V and PostV. (A) Model depicting transcriptional distances (the level of expression change between stages) for GS, PS, and RS berries from V to PostV; and transcriptional distances between GS and PS, and between PS and RS stages of the véraison cluster stage. Double-headed arrows **a** and **b** indicate fold changes in expression that were observed between GS and PS, and between PS and RS berry classes, respectively at V. ‘X, Y, and Z observed’ depict the observed fold changes in expression between V and PostV stages of GS, PS, and RS berry classes, respectively; ‘X calculated’ is the value computed as the sum of **a**, **b**, and Z; ‘Y calculated’ is the value computed as the sum of **b** and Z. (B, C) Linear correlations between the observed and calculated values of X (*R* ^2^=0.95), and the observed and calculated values of Y (*R* ^2^=0.96). The observed and calculated transcriptional changes in differentially expressed genes from all three tissues are shown together in the plots (Supplementary Worksheet 2A at *JXB* online). (This figure is available in colour at *JXB* online.)

Expression data of the three berry classes at V and PostV were used to assess for each gene qualitative and quantitative expression changes between berry transitional ripening stages; these changes were described as ‘transcriptional distances’. Along with the transcriptional changes between véraison transitional stages (from GS to PS to RS), the integration of their respective PostV expression data in the analysis revealed the overall transcriptional changes from a given véraison physiological stage to PostV in each berry class (Fig. 3A). It is clear that upon the transition of GS berries to their PS and RS stages, the remaining transcriptional distance for them would be same as that observed for the original PS and RS berry classes, considering that all three classes show synchronized transcriptional states at PostV. Therefore, the observed V to PostV transcriptional distance within the GS class was compared with the calculated transcriptional distance (the sum of the expression level changes between GS and riper berry classes at véraison; and the expression level changes within riper berry classes from V to PostV). The correlation between observed and calculated distances for the 3200 differentially expressed genes was linear, with *R* ^2^ >0.95 (Fig. 3B, C; Supplementary Worksheet 2A at *JXB* online). This indicates that GS, PS, and RS represent well-defined physiological ripening stages, each with an associated and distinct transcriptional state, and that a defined transcriptional programme exists between the given véraison to mature physiological stages regardless of the berry class. Given that all berry classes have the same amount of time (42 d) to reach maturity, the transcriptional distance that under-ripe berries need to cover must occur at a greater rate than in riper berries.

### Progression of physiological ripening among berry classes and enhanced ripening rate in under-ripe berries

**Fig. 4.**
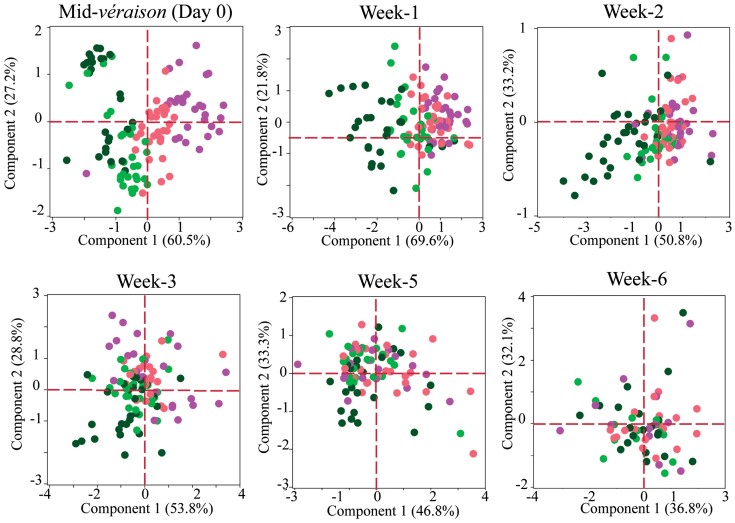
Reduction in ripening differences between GH, GS, PS, and RS berry classes from mid- véraison (V) to 6 weeks past mid-véraison. Principal component analyses performed using the colour index (Carreno *et al.*, 1995), total soluble solid content (TSS), and elasticity (Thomas *et al.*, 2008) of individual berries from GH (*n*=30), GS (*n*=30), PS (*n*=30), and RS (*n*=30) classes collected from six plants (one berry each from five clusters in each plant) at six time points. In the week 5 and 6 plots, *n* ≥30 for each berry class. TSS, colour, and elasticity data for individual berries from each of the four ripening classes collected at each time point were plotted. Dots representing GH, GS, PS, or RS berries are clustered from left to right, respectively. Loading plot data of the PCAs are given in Supplementary Table S6 at *JXB* online. (This figure is available in colour at *JXB* online.)

Monitoring the progress of physiological ripening parameters at a 7 d interval from V to PostV showed that accumulation of sugar and pigments continued until week 6 in all the berry classes and, at the same time, the ripening differences were greatly reduced by week 5 (Fig. 4). This suggests that the observed reduction in the ripening differences among berries of a cluster is not due to advanced berries reaching the limits of their accumulation with maturity, but rather is a result of enhanced accumulation in under-ripe berries (Fig. 4; Supplementary Fig. S3 at *JXB* online). PCAs of weekly ripening changes for each berry class relative to that of riper red berries indicate that rapid reduction in the ripening differences occurs between V and 3 weeks past V (Supplementary Fig. S4A–C). At the same time, the ripening in the majority of berries of each véraison berry class progressed together with time, as indicated by the discriminant analysis scores (Supplementary Fig. S4D–G), showing that differences between individual berries of a class do not contribute to the overall differences between classes. Further, if sugar and colour accumulation rates are expected to slow down progressively with the advancement of ripening, the under-ripe berries, upon turning red soft, would show the stage-appropriate slow down and hence maintain the véraison differences at week 6, which was not the case.

**Fig. 5.**
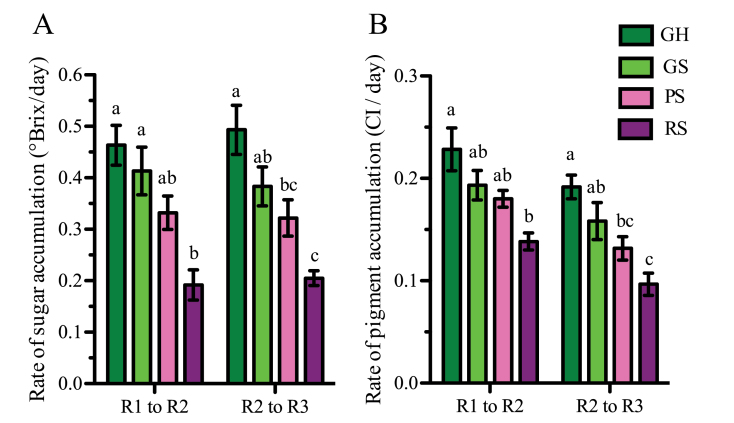
Rates of sugar (A) and pigment (B) accumulation in each berry class during a common ripening window across all the classes. R1, R2, and R3 represent reference stages at which total soluble solids (Brix)/colour index were similar across the four berry classes. The times required for each class to reach the R1–R3 stages (Table 1), derived from regression analyses, were used to calculate the rates of accumulation of sugar and colour between the physiologically equivalent reference stages (Supplementary Text at *JXB* online). The bars represent the mean of the rates from six plants (±SEM). Different letters denote significant differences (Tukey’s HSD, *P*<0.05). (This figure is available in colour at *JXB* online.)

To compare the relative rate of ripening progression in under-ripe and riper berry classes, the accumulation rates of sugar and colour were determined between the three common physiological ripening stages (R1–R3) across berry classes. In under-ripe berry classes, the physiological sugar and colour stages that were similar to those of mid-véraison RS berries were identified, which would be later than V (Supplementary Fig. S3 at *JXB* online). From these physiologically similar ripening stages, regression curves predicting the progression of sugar and colour were plotted for each berry class and used to derive times required for each berry class to reach the reference sugar and colour levels of those physiological stages (Table 1; Supplementary Worksheet 2B at *JXB* online). At these reference stages, sugar and colour in GH, GS, and PS were at the levels characteristic of RS berries at mid-véraison (R1), and at 1 (R2) and 2 weeks (R3) past mid-véraison. The calculated times to reach the successive sugar and colour levels of these stages were highly correlated (*R* ^2^ >0.72), which indicates that these two parameters progress concomitantly through the observed ripening transitions (Table 1). During this period, calculated accumulation rates for sugar and colour in under-ripe berries were significantly higher when compared with those of the next ripest berry class (Fig. 5; Supplementary Text). Trends of progression (Supplementary Fig. S3) indicate that enhanced ripening in under-ripe berries probably begins before the berries reach the R1 stage, although this cannot be directly ascertained. PS, GS, and GH berries lagged behind RS berries by 7, 10, and 13 d, respectively, at the R1 stage, but by the R3 stage they only lagged by 1, 3, and 5 d (Table 1). This enhanced ripening progression rate resulted in a significant reduction of variation in sugar content among berry classes from 5 °Brix at V to 1.2 °Brix at PostV.

**Table 1.** Days past mid-véraison at which under-ripe berries were at successive °Brix and colour levels of red berry reference stages

Reference ripening stage^*a*^	Reference °Brix/ colour value^*b*^	Days past mid-véraison (0)^*c*^			
		RS	PS	GS	GH
R1-Brix	12.6 (±0.3)	0	6.5 (±1.0)	9.9 (±1.0)	13.0 (±0.8)
R2-Brix	13.9 (±0.2)	7	10.8 (±1.1)	13.2 (±1.0)	16.1 (±1.0)
R3-Brix	15.3 (±0.2)	14	15.4 (±1.4)	17.0 (±0.7)	19.1 (±0.9)
R1-Colour	3.4 (±0.1)	0	4.3 (±0.7)	8.1 (±0.8)	12.7 (±1.1)
R2-Colour	4.4 (±0.1)	7	9.9 (±0.6)	13.4 (±0.9)	17.1 (±0.9)
R3-Colour	5.1 (±0.1)	14	15.0 (±0.5)	17.8 (±0.8)	20.8 (±0.9)

^*a*^ R1, R2, and R3 are specific Brix (total soluble solids) and colour index (CI) reference stages of RS berry class at mid-véraison (R1), and 1 (R2) and 2 weeks (R3) past-mid-véraison, respectively. The same ripening stages were reached at later times by under-ripe berries. Those times were determined through regression analyses (see Supplementary Worksheet 2B at *JXB* online) of the weekly increment data in sugar and pigment of each berry class separately. This enabled the comparisons of the days required between fixed sugar and pigment levels across berry classes.

^*b*^ Total soluble solid content (Brix) and colour index values in RS berries at the reference stages. Following their respective rates of sugar and pigment accumulation in each of the under-ripe berry classes, the days they would take to traverse through these reference stages were calculated.

^*c*^ Days at which each under-ripe berry class would reach the RS reference Brix and colour levels (see Supplementary Worksheet 2B at *JXB* online). The days taken by RS reference berries that were recorded are in the first column. Data are the mean of six plants with five berries of each ripening class contributing to the value of each plant. Errors bars represent ±SEM.

### Differential ripening-related hormone dynamics at equivalent ripening stages

**Fig. 6.**
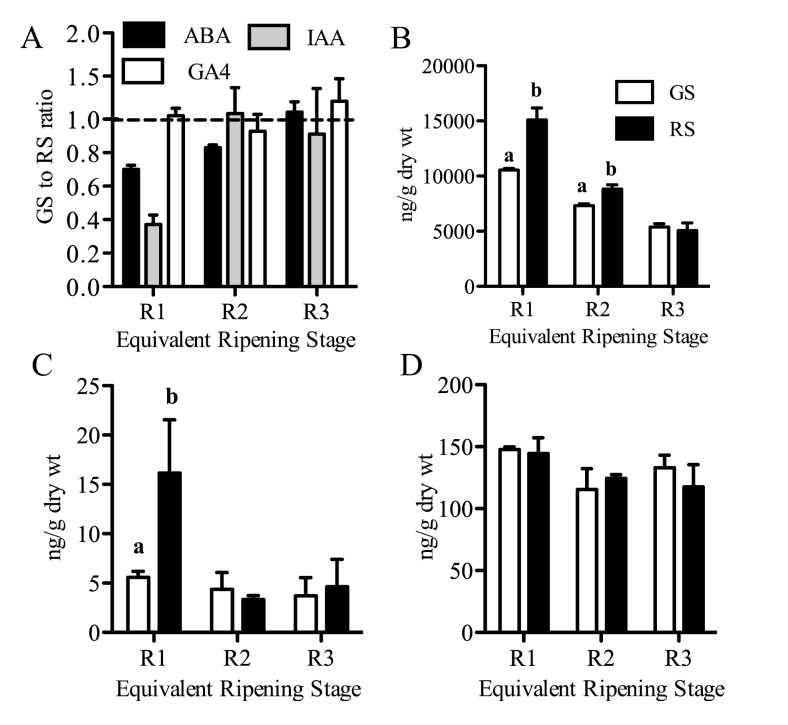
Differential hormone levels in GS and RS berry classes at the equivalent ripening stages. (A) Relative levels of ABA, IAA, and GA_4_ in pulp presented as the GS to RS ratio at equivalent ripening stages of R1, R2, and R3. (B–D) Absolute quantities of ABA, IAA, and GA_4_. Error bars indicate ±SEM of three replicate plants. Bars labelled with different letters are significantly different (Tukey’s HSD, *P*<0.05).

Levels of the two main hormones, ABA and IAA, known to promote and delay ripening, respectively (Böttcher *et al.*, 2010b; Ziliotto *et al.*, 2012), were quantified in GS and RS berries at the reference stages R1, R2, and R3, described above, for which sugar and colour levels in the two berry classes are within a very close range but at different times (Supplementary Table S5 at *JXB* online). The objective was to determine whether hormone concentrations were similar in both berry classes at these common physiological stages (Fig. 6A). GA_4_, a bioactive gibberellin in grape (Boss and Thomas, 2002), was used as a control because gibberellins act mainly during the early phases of berry development before ripening onset (Davies and Böttcher, 2009). The level of ABA was significantly different between the berry classes at R1 and R2 and that of IAA at R1 (Fig. 6B, C), while GA_4_ levels were similar (Fig. 6D). The R1–R3 stages encompass the phase of initial decrease in ABA from its highest level and the fully declined phase in auxin levels (Zhang *et al.*, 2003; Deluc *et al.*, 2009; Bottcher *et al.*, 2010b). ABA levels were 30% and 17% lower in under-ripe berries at R1 and R2, respectively, and higher at R3 (5%), indicating different ABA dynamics, including the decline rate through the stages, from those observed in riper berries (Fig. 6B).

### Differential gene expression levels and progression of transcription during equivalent ripening stages

**Fig. 7.**
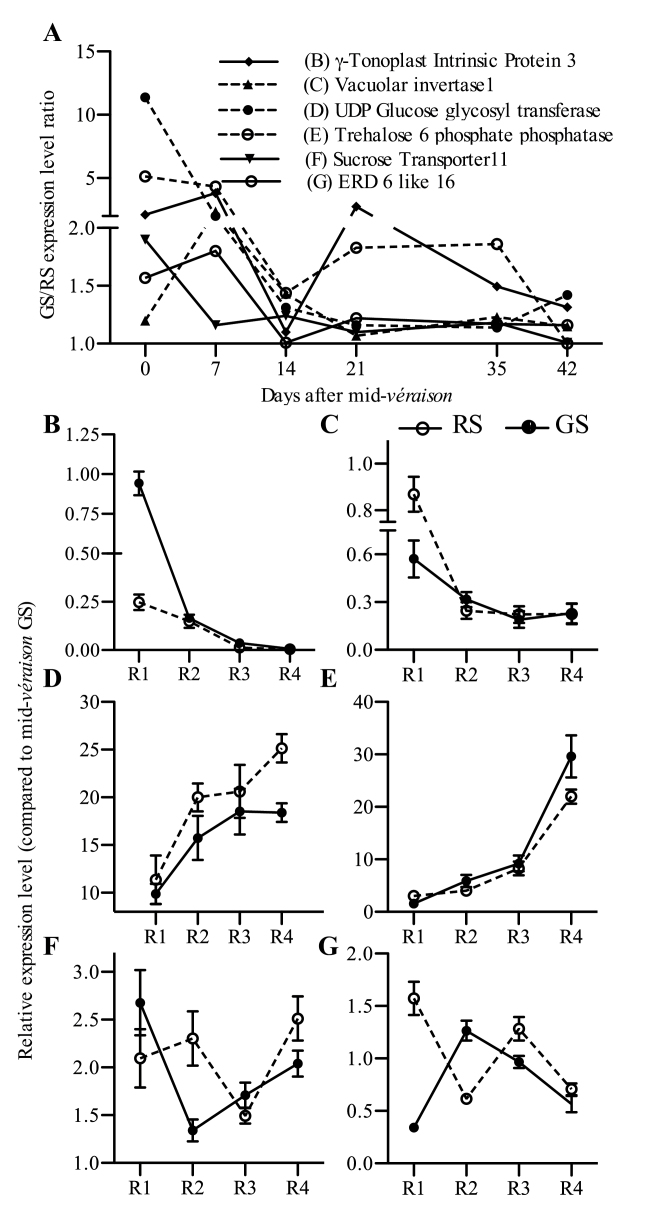
Differential gene expression dynamics in GS and RS berry classes. (A) Relative expression levels of six genes in GS and RS berries measured at mid-véraison (V) and at 7, 14, 21, 35, or 42 d after V expressed as the GS to RS ratio. (B–G) Gene expression levels and progression of transcription of the same genes in GS (filled circles) and RS (open circles) berry classes at R1, R2, R3, and R4 equivalent stages (Supplementary Table S5 at *JXB* online). Error bars indicate ±SEM of six replicates. Expression of the genes was measured in pulp tissue. See Supplementary text at *JXB* online for Gene IDs.

The progress of transcription and the reduction of expression variance between under-ripe and riper berries were examined for some genes related to sugar metabolism and transport at 0, 1, 2, 3, 5, and 6 weeks past mid-véraison in GS and RS berry classes (Fig. 7A). High véraison stage variance that was observed in the transcriptomic data of 2010, which was one of the criteria in the selection of genes, was not as pronounced in the experiments conducted in 2011. This could be due to the differences in the véraison stage assessment between the years, but synchronization of the gene expression levels towards maturity was evident (Fig. 7A). Expression of three genes indicated maximum RVs at week 2, while two other genes synchronized at week 3 (Fig. 7A). Observation of the expression dynamics for these genes in GS and RS berries across their equivalent ripening stages (R1–R4, Supplementary Table S5 at *JXB* online) showed that the levels of expression or progress between the physiologically equivalent stages were different in these berry classes. If the expression dynamics of RS berries is considered the normal course during berry development, then the readjustment in under-ripe berries probably occurred through an adjusted transcription rate (Fig. 7B–E) or altered expression pattern (Fig. 7F, G). Unlike the accumulation of metabolites such as sugars, pigments, and hormones, gene expression is more subject to many transient changes. However, based on the transcriptional distance model (see Fig. 3), the observed transcriptional modulations in under-ripe berries are imperative for synchronization to occur.

### Identification of ripening-related transcriptional modules and active regulatory motifs

**Fig. 8.**
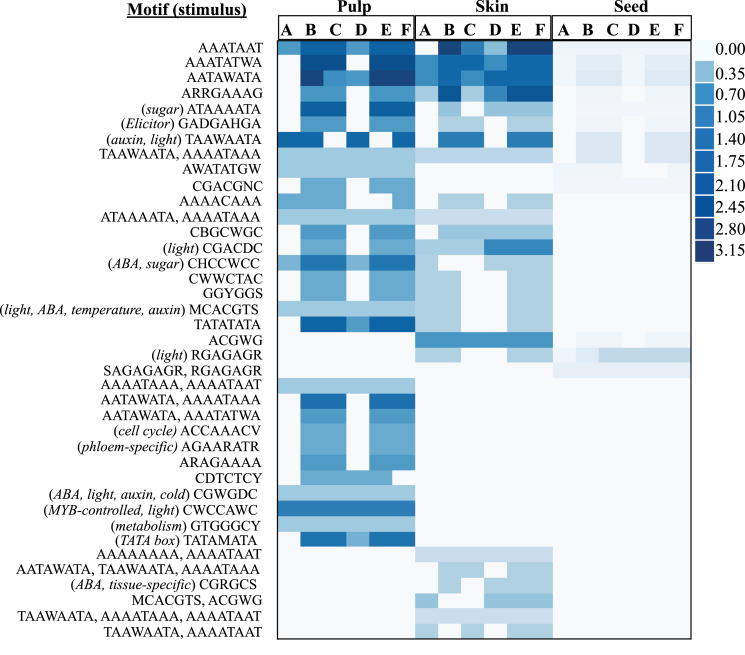
Heat map of functionally active regulatory motifs during the stages of ripening in pulp, skin, and seed tissues. Rows are regulatory motifs or a combination of motifs for which DISTILLER detected transcriptional modules. Known stimuli for which the motifs were reported to respond are given in parentheses. Conditions A to F represent the increase/decrease of gene expression between pre-véraison and green soft (A), green soft and pink soft (B), pink soft and red soft (C), red soft and post-véraison green soft (D), post-véraison green soft and post-véraison pink soft (E), and post-véraison pink soft and post-véraison red soft (F). Each entry indicates the extent of activity of the motif in a specific condition. Darker entries correspond to the most activity as a part of most transcriptional modules and the most number of genes. (This figure is available in colour at *JXB* online.)

Data from differentially expressed genes between véraison ripening classes were analysed using a module-based inference method (DISTILLER) to identify ripening-related co-expressed genes with shared over-represented regulatory motifs (transcriptional modules) and a conditional specificity (Lemmens *et al.*, 2009), where transitional ripening stages were regarded as conditions. Forty-eight significantly enriched putative regulatory motifs were identified in the data set, the majority of which were responsive to sugar, auxin, ABA, ethylene, light, and temperature, which have established roles in ripening (Supplementary Worksheet 2C at *JXB* online). The linearized sequence of pre-véraison to maturity stage expression dynamics and the motif data were used as input for DISTILLER. The 50 most significant transcriptional modules recovered by the analysis identified 167, 149, and 78 genes with 32 of the input motifs in pulp, skin, and seed, respectively (Supplementary Worksheets 2D–F). Most enrichment of transcriptional modules was found between the GS–PS, PS–RS, GSH–PSH, and PSH–RSH transitional stages (conditions) (Supplementary Worksheets 2D–F). As DISTILLER looks for genes with a similar extent and trend of expression changes between two ripening stages, every module active in any of the véraison conditions of A, B, C, and D, must also be active in E and F, which are maturity ripening stages. Since the transcriptional states of the three berry classes were synchronized, the two criteria of extent of change in expression and trend, which is close to zero in all genes, are always met in these conditions (Supplementary Worksheets 2D–F). In terms of the ripening stage-dependent relevance of the motifs, conditions encompassing GS to RS ripening stages at véraison were found to be enriched with a number of active motifs as part of transcriptional modules, and several motifs were uniquely active in specific tissues during the ripening process (Fig. 8).

## Discussion

Initiation of the ripening for most individual berries of a cluster precedes the grape cluster developmental stage of véraison by several days. Different ripening classes found at this stage have distinct ripening characteristics and are at various stages into their ripening programme (Lund *et al.*, 2008). It was observed that individual berries within each class undergo quantitatively similar physiological changes between transition stages, until maturity, demonstrating a well-defined programme of ripening, from initiation to maturity, which is followed by all the berries. Moreover, the observation showing that all classes, in spite of initial differences, reach similar ripeness levels at maturity indicates that a post-véraison regulatory mechanism for ripening synchronization among the berries of a cluster is possible.

The status of the sugar content and hormone dynamics are believed to signal ripening initiation, and import of sugars depends on the relative sink activity of the berries, which is influenced by factors such as limitation of the source, sink size, and metabolic activity (Ho, 1988). Cell wall invertases, sugar transporters, and sugar-degrading enzymes are part of the molecular processes that determine sink strength, and their activities can be dynamically altered during berry development (Zrenner *et al.*, 1995; Herbers and Sonnewald, 1998; Balibrea *et al.*, 2003). In a system of multiple competing sink units in the same transport system as in a grape cluster, transport conductivity and relative sink strengths of individual berries is an important consideration (Bustan, 1995). The differences in sugar accumulation rates show under-ripe berries as stronger sinks compared with riper berries during their equivalent ripening stages. Decreased sink strength and metabolic activity during advanced ripening as seen in riper berries is possible, but the sink strength of under-ripe berries did not decrease to the stage-appropriate level even when they reached the advanced ripening stage. Reprogramming of the ripening-related transcriptome of the berries might occur when the competing sink units cease to be strong sinks (Pastore *et al.*, 2011). Furthermore, the rate of sugar accumulation might influence several sugar-sensing pathways as sugars act as signal molecules (Hanson and Smeekens, 2009; Smeekens *et al.*, 2010; Eveland and Jackson, 2012).

In grape, ripening onset is marked by rapid transcriptomic and hormonal changes (Davies and Böttcher, 2009), and the events of cell wall loosening, sugar loading, and pigment accumulation depend mainly on the decrease in auxin levels coupled with the increase in ABA levels (Davies and Böttcher, 2009; Gambetta *et al.*, 2010; Castellarin *et al.*, 2011; Ziliotto *et al.*, 2012). Different dynamics of IAA and ABA observed in under-ripe berries from that of riper berries at physiologically similar stages is expected to impact the expression of many ripening-related genes. In both climacteric and non-climacteric fruits, most ripening changes rely on a fine balance between hormones, and their concentrations at specific developmental stages affect various metabolic pathways (McAtee *et al.*, 2013). Application of 1-naphthaleneacetic acid (NAA), a synthetic auxin analogue, during the pre-véraison stage was shown to cause changes in the expression of auxin- and ABA-related genes, a delay in ripening initiation, an increased post-véraison growth rate, and a reduced variance in sugar levels among berries of a cluster (Böttcher *et al.*, 2010a; Ziliotto *et al.*, 2012). It is conceivable that NAA application perturbs the natural decline of IAA in advanced berries and delays the ripening, thereby reducing variance between under-ripe and riper berries. In this context, a differentially timed decline of IAA and its possible influence on ABA dynamics in under-ripe berries may be a factor in the enhanced ripening.

The ‘transcriptional distance’ model shows defined transcriptional programmes between specific physiological ripening stages. The expression variances observed between under-ripe and riper berries of mid-véraison clusters reflect the transcriptional distances between these green, pink, and red stage transitions, and the transcriptional programme of under-ripe berries lagged by 7–10 d. Given the same developmental duration for all the berry classes, the extent of reduction in the expression variance is an indicator of the level of altered transcription of a given gene through either rate or pattern changes. Such plastic transcriptional processes and growth adjustments in response to water deficit, salt stress, and environmental changes are common in plants (Costantini *et al.*, 2008; Tardieu *et al.*, 2011). Transcriptome reprogramming in grape berries that were subjected to different viticulture practices and environmental conditions was reported (Dal Santo *et al.*, 2013). In the present data, several genes that respond to environmental conditions, carbohydrate import and metabolism, and hormones showed high reduction of expression variances. Incidentally, many regulatory motifs, known to respond to stimuli including light, ABA, IAA, sugar, and other nutrients, were found enriched in the data. These observations support the assumption that ripening synchronization is achieved through discrete changes as part of the regular ripening programme.

A study by Dal Santo *et al.* (2013), which emphasizes the plasticity of ripening-related gene expression, identified genes that are developmentally regulated but lack expression plasticity during the progress of ripening under different environmental conditions (Dal Santo *et al.*, 2013). Similarly, the relative reduction of expression variances of genes observed in the present study indicates their importance in the programme of berry ripening. As a general practice, transcriptomic and metabolic approaches relate the expression variance between developmental stages to the functional relevance of the genes (Deluc *et al.*, 2007; Pilati *et al.*, 2007; Ali *et al.*, 2011; Guillaumie *et al.*, 2011; MartinezEsteso *et al*., 2011; Lijavetzky *et al.*, 2012). However, the assessment based on the extent of reduction in variance eliminates a large number of developmental-related genes that may not necessarily be ripening related. Further, the stage-specific importance of gene networks can be ascertained through the model of the transcription programme such as pigment biosynthesis-related genes that are expected to be most active during green, pink, and red stage transitions of véraison (Ali *et al.*, 2011; Castellarin *et al.*, 2011). These genes are expected to synchronize during early post-veraison, when green berries reach the physiological red berry stage. Most genes with unaltered expression from véraison to maturity in riper berries can be classified as of early post-véraison importance. In the case of genes that continue to have significant expression changes towards maturity, stage-specific relevance is obscured and would need additional time points to be identified.

The asynchronous nature of the ripening initiation in a grape cluster may arise from differences in flowering times and fertilization events (Olimpieri *et al.*, 2007; Dorcey *et al.*, 2009), or may depend on the relative physiological readiness of the fruits (Joas *et al.*, 2011). In several plants, primigenic dominance of earlier developed fruits plays a role in ripening differences among fruits (Stephenson *et al.*, 1988; Bangerth, 1989; Bangerth *et al.*, 2000). Apart from the reasons for asynchronous ripening initiation, it was demonstrated that differential progression of the ripening programmes among berries of a cluster, along with readily quantifiable physiological stages, offers a useful comparative system to identify genes involved in the ripening process. Cluster-level regulation such as berry to berry hormone signals and/or other mobile signals that communicate the relative status of sink activities might be possible in the synchronization to achieve uniform cluster ripening. However, it would be of great significance to identify the developmental triggers that stimulate the ripening programme most.

## Supplementary data

Supplementary data are available at *JXB* online.

Supplementary text

Figure S1. PCA plot of skin, pulp, and seed tissues of berry classes at mid-véraison (V) and 5-weeks post-véraison (H) stages according to their normalized expression.

Figure S2. Relationship between ‘level of expression’ and ‘expression variance among berry classes’ at mid-véraison (V) and 5-weeks post-véraison (PostV).

Figure S3. Increases in sugar and pigment accumulation monitored at 7 d intervals from mid-véraison (0) until maturity (day 42) in GH, GS, PS, and RS berries.

Figure S4. Ripening progress in GH, GS, and PS berry classes in relation to that of the RS berry class.

Table S1. Evaluation of variances in ripening parameters of berries from different clusters and experimental plants to green hard, green soft, pink soft, and red soft ripening classes.

Table S2. Discriminant analyses showing the percentage of berries from each berry class sampled as GH, GS, PS, or RS berries (rows) assigned to ripening classes (columns).

Table S3. Percentages of genes with specific trends in expression during the transition from V to PostV in high- and low-RV gene sets.

Table S4. Ripening-associated genes identified using reduction in variance as the selection criterion.

Table S5. Ranges of TSS and colour index values in GS and RS berry classes at equivalent ripening stages.

Table S6. Component loadings of the principle component analyses.

Worksheet S1A. Differentially expressed genes at véraison (V).

Worksheet S1B. The level of variance at V and PostV and the range of reduction in variance from V to PostV.

Worksheet S1C. Differentially expressed genes at 5 weeks post-véraison.

Worksheet S1D. Variation in the expression values of the replicates.

Worksheet S1E. Single enrichment analysis for enriched GO terms.

Worksheet S2A. Observed and calculated transcriptional distances (log and fold change) between and within berry classes of the DGE

Worksheet S2B. Workflow followed to calculate the times for under-ripe berries to reach Brix and colour levels of the three reference stages.

Worksheet S2C. List of over-represented words and motifs detected by DREME.

Worksheet S2D. Enriched modules of genes during different phases of ripening detected by DISTILLER in pulp.

Worksheet S2E. Enriched modules of genes during different phases of ripening detected by DISTILLER in skin.

Worksheet S2F. Enriched modules of genes during different phases of ripening detected by DISTILLER in seed.

Supplementary Data
