# Supplementary material for: A comparative study of ripening among berries of the grape cluster reveals an altered transcriptional programme and enhanced ripening rate in delayed berries
Source: J Exp Bot. 2014 Aug 18;65(20):5889–902. doi: 10.1093/jxb/eru329 (PMC4203125; doi:10.1093/jxb/eru329)
Supplement: Supplementary Data [file supp_65_20_5889__index.html]

A comparative study of ripening among berries of the grape cluster reveals an altered transcriptional programme and enhanced ripening rate in delayed berries — A comparative study of ripening among berries of the grape cluster reveals an altered transcriptional programme and enhanced ripening rate in delayed berries — A comparative study of ripening among berries of the grape cluster reveals an altered transcriptional programme and enhanced ripening rate in delayed berries — Supplementary Data 

# A comparative study of ripening among berries of the grape cluster reveals an altered transcriptional programme and enhanced ripening rate in delayed berries

## Supplementary Data

Data files

**Files in this Data Supplement:**

- Supplementary Data - Supplementary Data
- Supplementary Data - Supplementary Data
- Supplementary Data - Supplementary Data
