# Supplementary material for: A comparative study of ripening among berries of the grape cluster reveals an altered transcriptional programme and enhanced ripening rate in delayed berries
Source: J Exp Bot. 2014 Aug 18;65(20):5889–902. doi: 10.1093/jxb/eru329 (PMC4203125; doi:10.1093/jxb/eru329)
Supplement: Supplementary Data [file supp_eru329_jexbot125237_file001.pdf]

A comparative study of ripening among berries of the grape cluster reveals altered transcriptional program and enhanced ripening rate in delayed berries

Satyanarayana Gouthu, Shawn T. O'Neil, Yanming Di, Mitra Ansarolia, Molly Megraw and Laurent G. Deluc

Supplementary text

Ripening progress in each class in relation to RS berries during the first 3-week period

To assess the progress of ripening during early-*véraison* in GH, GS, and PS berries relative to that of RS berries, principle component analyses were performed using TSS, color index, and elasticity data of individual berries from each under-ripe berry class along with those of RS berry class during the first 3-week period following mid-*véraison* (Supplementary Fig. S3A-C). No ripening progress was observed in GH berries between V and Week 1 (Supplementary Fig. S3A). In contrast, the ripening progress of GS berries was apparent at V but still lagged behind RS berries at Week 3 (Supplementary Fig. S3B). PS berries matched the RS berry ripening state by Week 1 and further progress was concomitant in both classes (Supplementary Fig. S3C). Discriminant analyses to examine the advancement of individual berries of a class (Supplementary Fig. S3D-G) showed that the majority of berries in a class advanced together during the first 3-week period, after which each berry class ceases to maintain its unique identity.

Rates of accumulation of sugars and pigments in berry classes

Increases in total soluble solids (TSS, °Brix) and color index [(180-h)/(C+L)] (Carreno and Martinez, 1995) were monitored in five clusters each of six plants to assess ripening progress during their equivalent ripening periods. GH, GS, PS, and RS berries were identified and tagged with color-coded strings. One berry from each ripening class per cluster was sampled every week and sugar and color data was obtained for each berry. At V, TSS and color index in RS berries were 12.6 and 3.4 respectively, levels that were observed one or two weeks later in GH, GS, and PS berries. Weekly increases in TSS and color index over a two-week period were plotted from these physiologically similar stages in each class. Equations to calculate the days required to reach a given TSS and

color stage at the ripening rate characteristic of each berry class were obtained by fitting second-order polynomial curves. Using the respective equations we could predict when GH, GS, and PS berries would reach the TSS and color levels observed in RS berries (Supplementary worksheet 2B). The TSS level of RS berries at V, and at 1 and 2 weeks past-V were designated as Brix/color stages of R1, R2, and R3 respectively. Although RS berries took seven days to traverse the increments between the stages, the times calculated for under-ripe berries to traverse the same increments were lower (Table 1).

RNA isolation and NimbleGen Vitis vinifera GeneChip Array Hybridization and data analysis

Total RNA was isolated from the skin, pulp and seed tissues of the pooled berries using the RNeasy Midi Kit (Qiagen Inc., Valencia, CA). Because of the high sugar and phenolic content of the tissues, Qiagen RLC buffer (2% polyethylene glycol (MW 20,000), 0.2 M sodium acetate (pH 5.2), and 1% β-mercaptoethanol) was substituted for lysis buffer. For the reminder of the procedure, the manufacturer's protocol was followed, including on-column DNase digestion (RNase-free DNase, Qiagen, Valencia, CA). First-strand cDNA was prepared from 10 μg total RNA using SuperScript III Reverse Transcriptase (Invitrogen, Carlsbad, CA) and oligo dT primers. The second-strand cDNA synthesis reaction contained 3 μg random primer, 5U Klenow fragment (New England Biolabs, Ipswich, MA), Klenow buffer, and 2.5 mM dNTP mixture. Following incubation at 37 °C for 1.5 h, the double-stranded cDNA was purified using MiniElute PCR Purification Spin Columns (Qiagen Inc., Valencia, CA). The double-stranded cDNA was labeled using the NimbleGen One-Color DNA Labeling Kit (NimbleGen, Madison, WI) following the NimbleGen Array user guide. A 2 μg sample of Cy3-labeled cDNA was hybridized to the microarray according to the manufacturer's instructions. NimbleGen Microarray 090818 Vitis exp HX12 (Roche, NimbleGen) contains 29,971 non-redundant probes in quadruplicate, based on annotations of the Grape 12X Assembly performed by The Institute of Applied Genomics (Italy). Hybridization and washes were performed using NimbleGen reagents and hardware, and microarrays were scanned with an Axon GenePix 4200A Pro Scanner, according to the protocols provided by NimbleGen. Quality control, normalization, and the signal-to-noise

ratio for hybridization signals for samples in V and PostV were performed using the web-based ANAIS tools (Simon and Biot, 2010). The Robust Multichip Average (RMA) normalization process involved background adjustment and quantile normalization (Irizarry *et al.*, 2003).

Quantitative Real-time RT-PCR analysis

RT-PCR was performed using ABI 7500 Fast Real-Time PCR system (Applied Biosystems). Briefly, 5-10 ng of cDNA was used as template for analysis using QuantiFast SYBR Green (Qiagen). Specific oligonucleotide primer pairs were designed with Primer 3 software (http://biotools.umassmed.edu/bioapps/primer3_www.cgi). Amplification of the single amplicon per PCR product was verified by analyzing dissociation curves. In this study, six *V. vinifera* reference genes were evaluated: gamma tonoplast aquaporin, 3 sucrose transporter 11, ERD6-like 16, vacuolar invertase 1, UDP-Glucose glycosyltransferase, and trehalose-6P phosphatase. Peptidyl-prolyl *cis-trans* isomerase (VIT_06s0004g06610) was used as a reference gene for normalization based on its low M value in our expression data (Vandesompele *et al.*, 2002). All PCR reactions were done with three biological and two technical replicates. The reaction conditions were: heat activation at 95 °C for 5 min (one cycle); 95 °C for 10 s, 60 °C for 30 s (40 cycles). Data were acquired and exported with the 7500 Fast Software version 2.0.6 (Applied Biosystems) and relative gene expression was calculated using the $^{\Delta\Delta}C_t$ method. Relative fold-expression for each gene was calculated, in which the level of expression in GS berries at V was set at 1.

Oligonucleotide sequences

Name	Vitis ID	Forward	Reverse
Gamma tonoplast aquaporin 3	VIT_06s0061g00730	ATCAGGGCCGTCCATTTTAT	AACGCCAATTACATGCATCA
Sucrose transporter 11	VIT_18s0001g08210	GGACCATGGGATCAACTTTT	AGATCGAGGAATAGCCAAGATG
ERD6-like 16	VIT_05s0020g02170	TTTTTGTGGCAAAGATCGTG	TCCGAGTACCATCTGCATGT
Vacuolar invertase 1	VIT_16s0022g00670	CCTCTACATTAGCTCCGTTTGG	CAGGACATGACAAAGGATTGAA
UDP-Glucose glycosyltransferase	VIT_00s0324g00050	GCTGTTGGTAGTTCACCTTGC	GGCAAAGATGGCTGTGACTT
Trehalose – 6P phosphatase	VIT_00s0233g00030	AATAAAGGTGGTGGGTGCTG	AGGCATTACAAGTGCCAAGG

Peptidyl-prolyl *cis*-*trans* isomerase	VIT_06s0004g06610	TCCACTCTTGCCTTTTTGCT	ACGGATCAAAGCCATTTCTG

Identification of transcriptional modules during the ripening transitions

The data integration framework, 'DISTILLER' was used to identify transcriptional modules (co-expressed genes sharing overrepresented *cis*-regulatory elements or motifs) in the data of differentially expressed genes between the berry ripening stages (Lemmens *et al.*, 2009). DISTILLER identifies condition-dependent expression modularity by integrating the overrepresentation score of motifs identified in the promoter region of analyzed genes and the expression dynamics of the same genes. Overrepresented motifs in the data were mapped and the Positional Weight Matrices (PWMs) representing these regulatory motif sites were extracted using DREME algorithm (Bailey, 2011). Nature of the stimuli that elicit responses from known regulatory motifs was extracted from PLACE database (Higo *et al.*, 1999). The PWMs are used to scan the 2 kb upstream promoter region of each gene in order to discover the putative binding sites of each regulatory element represented by a PWM. A log-likelihood-based score compares the probability of observing a particular subsequence in promoter region according to the PWM model to the probability of observing that subsequence according to a background model. The scanning is performed with the Scanner Toolset for Transcription Factor Binding Site Discovery (http://megraw.cgrb.oregonstate.edu/software/TFBS-Scan/) (Megraw *et al.*, 2013). A high score is indicative of a good match to the TF binding motif. The motifs form a binary matrix in which the columns correspond to each motif and rows indicate each gene; motifs found to have at least one putative binding site in the promoter region of a gene receive value 1, otherwise 0. This binary matrix is used as an input to DISTILLER for identifying transcriptional modules. Along with the GS, PS, and RS datasets of mid-*véraison* and maturity cluster stages, we included gene expression data of berries of pre-*véraison* cluster that comes before GS stage, forming a linear sequence of seven developmental stages (PV, GS, PS, RS, GSH, PSH, RSH), to increase the statistical strength of the DISTILLER analysis. Expression differences of each gene between two successive ripening stages was assigned to conditions A, B, C, D, E, F. For example, condition A has the expression difference of a gene from pre-*véraison* (PV) to

green soft stage (GS), which contained the information on the direction of gene expression (up or down-regulation), and the extent of expression differences between the stages. To identify the relative functional activity of motifs across the conditions, overall activity scores were assigned for each motif based on the total number of modules it belongs to and the number of genes in those modules. Similar scores for motifs in each condition were assigned, and integrated with the overall activity score, which was normalized across the motifs. So the final activity scores reflect the relative activity of each motif between tissues, conditions, and across the motifs.

REFERENCES

Bailey TL. 2011. DREME: motif discovery in transcription factor ChIP-seq data. Bioinformatics (Oxford, England) **27**, 1653–1659.

Carreno J, Martinez A. 1995. Proposal of an index for the objective evaluation of the color of red table grapes. Food Research International **28**, 373–377.

Higo K, Ugawa Y, Iwamoto M, Korenaga T. 1999. Plant cis-acting regulatory DNA elements (PLACE) database: 1999. Nucleic acids research **27**, 297–300.

Irizarry RA, Hobbs B, Collin F, Beazer-Barclay YD, Antonellis KJ, Scherf U, Speed TP. 2003. Exploration, normalization, and summaries of high density oligonucleotide array probe level data. Biostatistics (Oxford, England) **4**, 249–264.

Lemmens K, De Bie T, Dhollander T, *et al.* 2009. DISTILLER: a data integration framework to reveal condition dependency of complex regulons in Escherichia coli. Genome biology **10**, R27.

Megraw M, Mukherjee S, Ohler U. 2013. Sustained-input switches for transcription factors and microRNAs are central building blocks of eukaryotic gene circuits. Genome biology **14**, R85.

Simon A, Biot E. 2010. ANAIS: analysis of NimbleGen arrays interface. Bioinformatics (Oxford, England) **26**, 2468–2469.

Vandesompele J, De Preter K, Pattyn F, Poppe B, Van Roy N, De Paepe A, Speleman F. 2002. Accurate normalization of real-time quantitative RT-PCR data by geometric averaging of multiple internal control genes. Genome biology **3**, 0034.1–0034.11.

Supplementary Table S1. Evaluation of variances in ripening parameters of berries from different clusters and experimental plants.

Comparison	Brix	Color index
ripening class	<0.0001	<0.0001
ripening class X cluster	0.8242	0.1748
ripening class X plant	0.0379	0.4959
ripening class X plant X cluster	0.4253	0.7181

The table displays the *p* values of ANOVA tests performed between different comparisons. Total soluble solids and color index of individual berry were used in the tests. Seventy-five berries of each ripening class that were used in the study were sampled from five different clusters of three experimental plants.

Supplementary Table S2. Discriminant analyses showing the percentage of berries from each berry class sampled as GH, GS, PS, or RS berries (rows) assigned to ripening classes (columns).

	**Mid-*véraison***				**5-Week Post- *véraison***			
	**GH**	**GS**	**PS**	**RS**	**GH**	**GS**	**PS**	**RS**
**GH**	69	31	0	0	21	32	21	26
**GS**	20	79	1	0	15	36	21	28
**PS**	0	4	93	3	14	20	34	31
**RS**	0	0	4	96	17	13	22	48

Color (L [lightness], h [hue angle], and C [chroma]), and total soluble solid content (°Brix) of individual berries were used as discriminant factors. Number of berries (n) is 75 for each class in the V plot; and 67, 61, 64, and 47 berries for GH, GS, PS, and RS respectively for PostV.

Supplementary Table S3. Percentages of genes with specific trends of expression from V to PostV ripening stages in high- and low-RV gene sets.

V to PostV expression*	Low RV set	High RV set
Up-regulated	22	40
Down-regulated	37	22
Plateau	41	38

*: Significance of the V to PostV expression was calculated using two-sided contrast analysis (See Material and Methods; Supplementary worksheet 1B)

Supplementary Table S4. Ripening associated genes identified using reduction in variance as the selection criterion

Gene ID	Gene Name	Tissue	RV[a]	Var[b]	Synch stage[c]
VIT_12s0034g01920	No hit	Pulp	30397	4.69	ES
VIT_01s0011g05930	S-adenosyl-L-methionine:carboxyl methyltransferase	Seed	14429	11.20	LS
VIT_12s0057g00940	Glucose-6-phosphate 1-dehydrogenase 2	Pulp	7875	0.44	ES
VIT_10s0003g01830	Aquaporin NIP1;2	Skin	7467	0.78	ES
VIT_18s0001g14350	No hit	Pulp	6608	10.81	LS
VIT_00s0394g00040	Alliinase EGF	Pulp	6606	6.99	ES
VIT_02s0025g04300	Thaumatin	Seed	5844	1.56	LS
VIT_13s0067g00660	Steroid 23-alpha-hydroxylase	Skin	5455	0.34	ES
VIT_07s0104g01710	HAK5 (High affinity K+ transporter 5)	Pulp	5194	2.50	LS
VIT_06s0004g06770	No hit	Skin	5085	0.38	LS
VIT_05s0051g00640	Purple acid phosphatase 23-ATPAP23/PAP23	Skin	4698	0.13	LS
VIT_00s0324g00050	UDP-glucose glucosyltransferase	Skin	3570	1.16	ES
VIT_07s0031g02560	UVB-resistance protein UVR8	Pulp	3431	1.62	LS
VIT_18s0001g14340	No hit	Pulp	3288	5.01	LS
VIT_13s0067g03820	Chalcone isomerase	Pulp	3192	0.75	ES
VIT_11s0016g04820	Agenet domain-containing protein	Skin	3189	1.01	ES
VIT_14s0128g00520	Alpha-L-fucosidase 2 precursor	Pulp	2377	0.72	ES
VIT_00s1286g00020	Elongation factor EF-G	Pulp	2310	0.06	LS
VIT_18s0001g11730	No hit	Skin	2133	0.66	LS
VIT_02s0154g00110	Trehalose-6-phosphate phosphatase	Pulp	2061	2.57	LS
VIT_12s0059g00320	EF hand	Pulp	1979	1.64	ES
VIT_18s0001g01130	Alpha-expansin 1 precursor	Seed	1943	0.69	LS
VIT_18s0001g00990	Calmodulin-domain protein kinase 9 CPK9	Pulp	1929	0.38	ES
VIT_18s0001g06390	Auxin-independent growth promoter	Pulp	1923	0.24	LS
VIT_15s0046g02400	Glycerol-3-phosphate acyltransferase 8	Pulp	1888	1.61	ES
VIT_07s0005g02370	Germin-like protein 2 [Vitis vinifera]	Seed	1765	6.49	LS
VIT_10s0003g00410	MLO6 (mildew resistance locus O 6)	Seed	1701	1.99	LS
VIT_03s0180g00110	Stress enhanced protein 1 (SEP1)	Pulp	1658	0.09	ES
VIT_03s0063g01290	Gibberellin 20 oxidase 2	Pulp	1637	1.03	ES
VIT_18s0001g15140	Unknown	Skin	1580	1.14	LS
VIT_04s0023g03790	Jasmonate methyltransferase	Skin	1555	4.65	LS
VIT_12s0059g00490	Unknown	Skin	1552	0.34	LS
VIT_08s0040g01840	No hit	Pulp	1552	1.43	LS
VIT_00s0615g00010	Cinnamyl alcohol dehydrogenase	Seed	1547	0.25	LS
VIT_18s0001g07610	EMB1674 (embryo defective 1674) kinase interacting family protein	Pulp	1365	0.82	ES
VIT_00s0260g00080	Ribosomal protein L32 60S	Skin	1292	0.05	ES
VIT_18s0001g11430	flavonoid 3-monooxygenase	Seed	1291	7.37	LS
VIT_13s0067g02930	Expansin [Vitis labrusca x Vitis vinifera] EXPA8	Seed	1277	0.67	LS
VIT_00s0865g00020	Unknown protein	Pulp	1253	3.25	LS
VIT_07s0031g01930	Myb TKI1 (TSL-kinase 1)	Skin	1185	1.22	LS
VIT_00s0225g00230	Alliin lyase precursor	Skin	1178	5.25	LS
VIT_13s0106g00280	CYP79A2	Pulp	1164	8.06	LS
VIT_12s0134g00160	Xyloglucan endotransglycosylase/hydrolase 16	Seed	1042	0.58	ES

VIT_08s0040g01600	TCP family transcription factor TCP11	Pulp	1025	0.92	LS
VIT_03s0017g00390	MADS-box protein SVP	Skin	960	1.35	ES
VIT_07s0031g00480	Protein kinase family	Pulp	953	0.60	LS
VIT_04s0023g01750	TVLP1	Skin	942	0.07	LS
VIT_13s0067g02300	Hypoxia-responsive	Pulp	936	2.17	ES
VIT_06s0004g02560	Kiwellin Ripening-related protein grip22	Seed	911	0.58	ES
VIT_11s0016g02530	Protein arginine N-methyltransferase	Pulp	911	0.23	ES
VIT_02s0025g04340	Osmotin	Seed	897	0.51	LS
VIT_18s0089g00160	1,4-beta-mannan endohydrolase	Skin	876	3.43	LS
VIT_17s0000g06880	Heparanase protein 2 precursor	Pulp	873	0.30	LS
VIT_05s0077g01880	DAG protein	Pulp	869	0.10	LS
VIT_03s0063g01310	Oxidoreductase, 2OG-Fe(II) oxygenase	Pulp	861	1.99	ES
VIT_12s0057g00700	Glucan endo-1,3-beta-glucosidase 3	Seed	849	0.28	LS
VIT_05s0051g00830	Dihydroxy-acid dehydratase	Pulp	819	0.04	ES
VIT_00s0684g00030	SEN1 (dark inducible 1)	Pulp	815	0.66	ES
VIT_01s0010g03390	Gag-pol polyprotein	Pulp	780	0.16	LS
VIT_03s0038g04330	Unknown	Seed	760	9.28	LS
VIT_18s0001g06390	Auxin-independent growth promoter	Skin	741	0.17	LS
VIT_03s0038g03410	NAC domain containing protein 36	Pulp	737	2.05	LS
VIT_15s0048g00500	Pectinesterase family	Pulp	733	3.53	LS
VIT_18s0001g06790	Ripening regulated protein DDTFR18	Seed	726	3.90	LS
VIT_12s0034g01910	Cupin family protein	Pulp	719	4.97	LS
VIT_01s0011g03670	Bifunctional nuclease	Skin	718	3.10	LS
VIT_19s0015g00140	Ribosomal protein S10 30S	Pulp	716	0.03	LS
VIT_18s0001g11490	CYP82C1p	Seed	706	3.67	LS
VIT_12s0034g01890	Cupin region	Pulp	702	4.90	LS
VIT_00s0394g00040	Alliinase EGF	Skin	699	6.11	LS
VIT_19s0015g02090	CYP72A59	Seed	682	0.50	LS
VIT_18s0164g00170	Diphenol oxidase	Seed	676	2.98	LS
VIT_10s0071g00320	GRAM domain-containing protein / ABA-responsive	Pulp	668	0.51	ES
VIT_18s0001g00950	Prolyl 4-hydroxylase alpha-2 subunit	Pulp	660	0.16	LS
VIT_18s0001g11520	Flavonoid 3-monooxygenase	Seed	645	4.14	LS
VIT_18s0001g15640	Pathogenesis-related	Seed	639	0.41	ES
VIT_06s0004g06030	Ca2+/calmodulin-regulated receptor kinase	Pulp	638	0.15	ES
VIT_01s0137g00520	CYP71B35	Skin	635	6.48	LS
VIT_16s0050g02400	Ethylene-responsive transcription factor Cytokinin response factor 4	Pulp	622	0.23	ES
VIT_16s0115g00120	Transformation/transcription domain-associated protein	Skin	617	1.63	LS
VIT_14s0066g01710	Leaf senescence protein	Pulp	615	2.40	LS
VIT_12s0142g00110	Splicing factor, arginine/serine-rich 2	Skin	596	0.59	LS
VIT_08s0007g04220	SER/ARG-rich protein kinase 4 SRPK4	Skin	595	0.06	LS
VIT_00s0276g00030	No hit	Skin	592	0.84	ES
VIT_02s0025g04340	Osmotin	Pulp	584	2.80	ES
VIT_05s0020g03200	Spermine synthase	Pulp	582	4.63	ES
VIT_18s0001g14340	No hit	Skin	572	5.17	LS
VIT_06s0080g00920	Photosystem I subunit O (PSAO)	Pulp	570	3.51	LS
VIT_12s0034g01900	Globulin-like protein	Pulp	566	5.27	LS
VIT_02s0025g03590	Phospholipid hydroperoxide glutathione peroxidase	Pulp	561	0.37	LS
VIT_18s0086g00680	No hit	Pulp	559	6.13	ES
VIT_18s0086g00320	No hit	Pulp	549	4.30	ES
VIT_14s0068g01400	UPF0497 family	Pulp	545	6.66	LS

VIT_00s2001g00010	No hit	Pulp	545	0.09	LS
VIT_00s0233g00030	Trehalose-6-phosphate phosphatase	Pulp	541	5.32	LS
VIT_08s0056g01230	Cycling DOF factor 2	Skin	540	0.30	ES
VIT_17s0000g08070	Aldehyde dehydrogenase 1 precursor	Pulp	533	0.31	ES
VIT_12s0034g01930	Globulin-like protein	Pulp	523	6.51	LS
VIT_18s0001g09710	MRG	Pulp	520	0.77	LS
VIT_18s0001g06720	Rieske [2Fe-2S] domain	Pulp	516	0.48	LS

Top 100 genes ranked in the order of their reduction in expression variance[a] (RV) from *véraison* to maturity among the berry classes. Marked in red are the 15% of the genes that were identified in the top 100 based on their *véraison to maturity*-expression differences[b] (Var) alone. Synch stage[c] denotes the timing of synchronization during the ripening progress assessed from *véraison* to maturity expression trend as early *véraison* stage (ES) and late maturity stage (LS) synchronizing genes (**Supplementary worksheet 1B)**. RV is calculated as the ratio between *véraison*- and maturity-stage expression variances. Very high numbers for RV numbers are due to very low to zero variance at maturity stage.

Supplementary Table S5. Ranges of TSS and color index values in GS and RS berry classes at equivalent ripening stages

Ripening stage	TSS range	Color index range
R1	11.5-12.5	3.2-3.3
R2	13.9-14.1	4.3-4.4
R3	16.0-17.2	5.0-5.5
R4	18.1-18.5	5.8-6.0

R1 to R4 are the physiological stages of similar ripening levels across all the berry classes. Total soluble solids and color index levels of mid-véraison-RS, and 1, 2, 3 weeks past mid-*véraison*, act as reference. Table shows the range of differences in the total soluble solid (brix) and color index for green and red soft berries at each considered stage. Under-ripe green berries were selected when they reach the closest reference levels for the purpose of comparing sugar and pigment accumulation rates, hormone levels, and gene expression dynamics between both the berry classes.

Supplementary Table S6. Component loadings of the principle component analyses.

A.
Eigen vector contributions to component scores of Fig. 1

Mid-*véraison* PCA

Vector	Component 1	Component 2	Component 3	Component 4
Brix	-0.50593	0.21194	0.82006	0.16316
L	0.50285	-0.44850	0.52883	-0.51607
C	0.46948	0.85782	0.10404	-0.18142
h	0.52035	-0.13448	0.19242	0.82106
Eigen value	86.1% (<0.0001*)	7.79% (<0.0001*)	3.47% (<0.0001*)	1.75% (NS)

Five week post-*véraison* PCA

Vector	Component 1	Component 2	Component 3	Component 4
Brix	-0.58008	0.25479	0.34371	0.69315
L	0.31605	0.87731	0.29713	-0.20532
C	0.58489	0.09655	-0.44204	0.67319
h	0.47065	-0.39509	0.77342	0.15560
Eigen value	44.1% (<0.0001*)	23.9% (<0.001*)	18.7% (<0.0308*)	13.3% (NS)

B. Eigen vector contributions to component scores of Fig. 4 PCAs

Mid-*véraison*, Day 0 PCA

Vector	Component 1	Component 2	Component 3
Elasticity	-0.30653	0.95175	0.01455
Brix	0.67167	0.22710	-0.70518
Color Index	0.67446	0.20638	0.70888
Eigen value	60.5% (<0.0001*)	27.2% (<0.0001*)	6.4% (NS)

Week 1 PCA

Vector	Component 1	Component 2	Component 3
Brix	0.59788	0.47081	-0.64875
Elasticity	-0.49845	0.85220	0.15910
Color Index	0.62777	0.22825	0.74418
Eigen value	69.6% (<0.0001*)	21.8% (<0.0001*)	8.6% (NS)

Week 2 PCA

Vector	Component 1	Component 2	Component 3
Brix	0.70531	0.03165	0.70819
Elasticity	-0.11115	0.99158	0.06638
Color Index	0.70013	0.12553	-0.70290
Eigen value	50.8% (<0.0001*)	33.2% (<0.0005*)	16.0% (NS)

Week 3 PCA

Vector	Component 1	Component 2	Component 3
Brix	0.65973	-0.08929	0.74618
Elasticity	-0.57865	0.57318	0.58020
Color Index	0.47950	0.81455	-0.32648
Eigen value	53.8% (<0.0001*)	28.8% (<0.0267*)	17.4% (NS)

Week 5 PCA

Vector	Component 1	Component 2	Component 3
Elasticity	-0.70732	-0.00252	0.70689
Brix	0.68926	-0.22443	0.68888
Color Index	0.15691	0.97449	0.16048
Eigen value	46.8% (<0.0049*)	33.4% (<0.0436*)	19.8% (NS)

Week 6 PCA

Vector	Component 1	Component 2	Component 3
Brix	0.66821	-0.24894	0.70109
Elasticity	0.31002	0.94981	0.04178
Color Index	0.67630	-0.18944	-0.71185
Eigen value	36.8% (NS)	32.1% (NS)	31.1% (NS)

Minimum weight of the vector scores to each component is zero. In the last row of each table, Eigen values for each component are given in percentage. *p*-values in parenthesis in the last row indicate the significance for variance between eigen values.

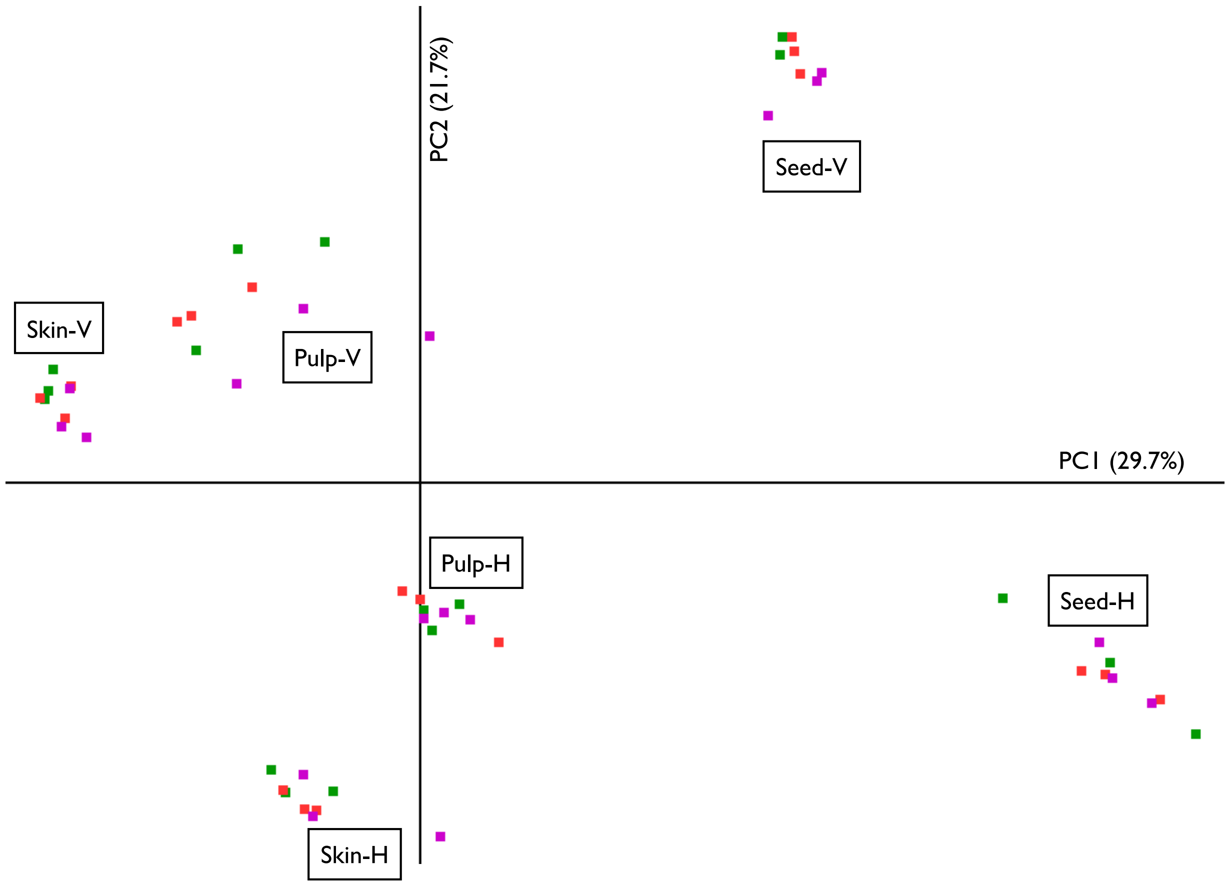

Supplementary Fig. 1. PCA plot of skin, pulp, and seed tissues of different berry classes from mid-*véraison* (V) and post-*véraison* (H) maturity stages according to their normalized expression. The assigned are Green for green soft; red for pink soft; and purple for red soft berries in each tissue and sampling times V and H.

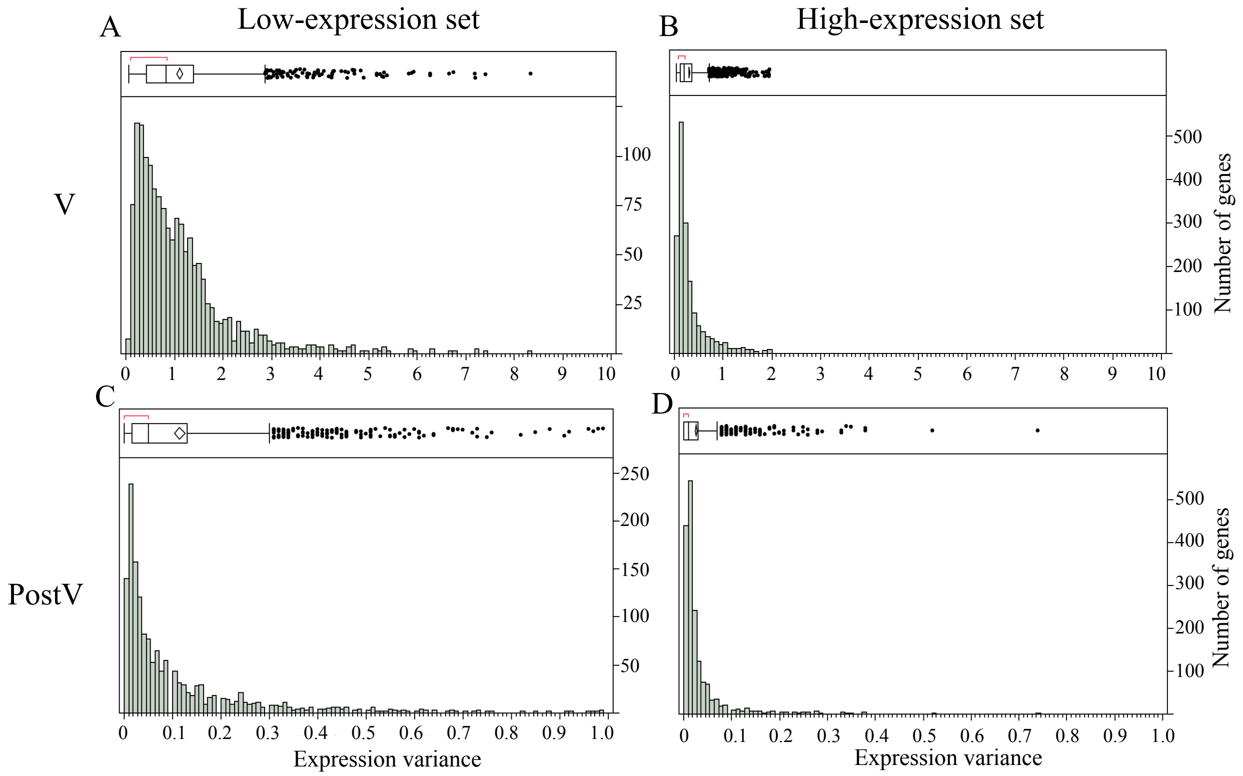

Supplemental Figure S2. Relationship between 'level of expression' and 'expression variance among berry classes' at mid-*véraison* (V) and 5-weeks post-*véraison* (PostV). Genes with expression value below and above 11 were grouped into (A and C) low (1505 genes) and (B and D) high (1694 genes) expression sets, respectively, and distribution of their expression variance is plotted in each set. Whiskers indicate the range of expression variance and the boxes indicate the 25, 50 and 75 percentile distribution with outliers shown as dots.

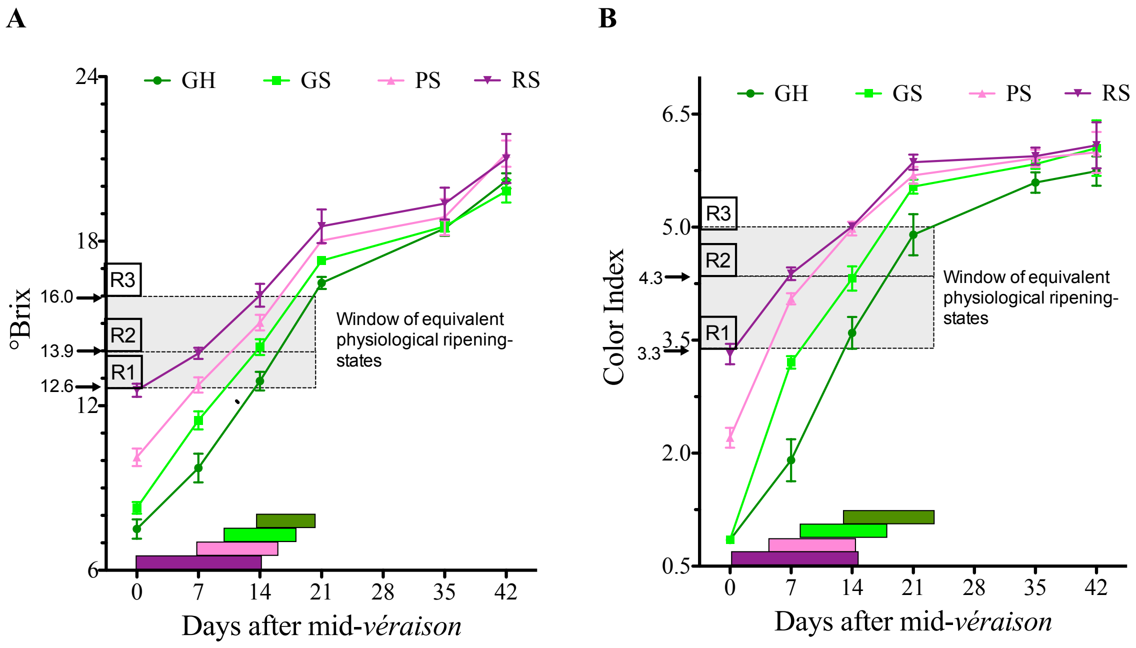

Supplementary Figure S3. Increases in sugar (*A*) and pigment (*B*) accumulation monitored at seven-day intervals from mid-*véraison* (0) until maturity (day 42) in GH, GS, PS, and RS berries. Data are mean of six plants where five berries per ripening class were sampled from five clusters of each plant. Errors bars represent ± SEM. R1, R2, and R3 represent reference stages at which total soluble solids (Brix)/color index across the four berry classes were similar and berries of all classes were at common physiological ripening stages. The width of the color blocks at the bottom part of the plots shows the length of time for each berry class to progress from R1 to R3 ripening stages.

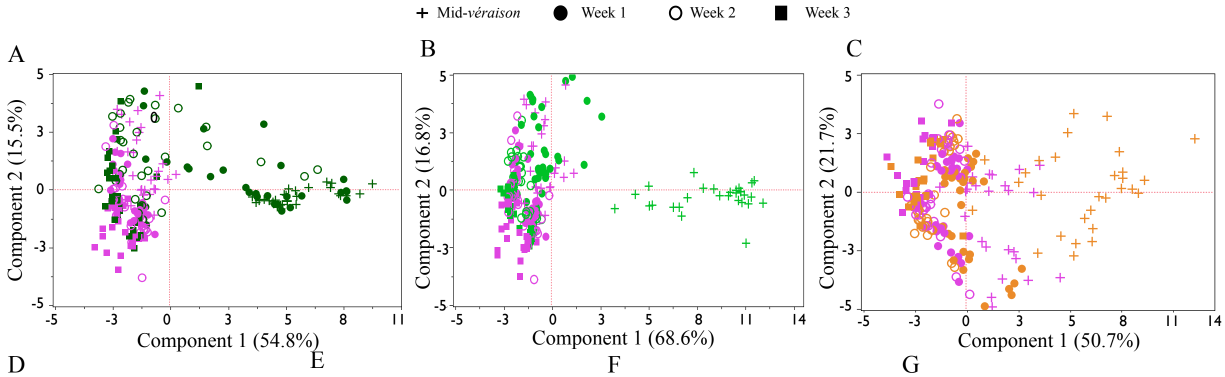

D

GH	MV	WK1	WK2	WK3	WK5	WK6
MV	93	6				
WK1	41	38	17	3		
WK2	3	17	63	13		
WK3			14	62	21	3
WK5				28	48	24
WK6				9	36	55

E

GS	MV	WK1	WK2	WK3	WK5	WK6
MV	100					
WK1		93	7			
WK2		7	90	3		
WK3			7	67	20	7
WK5				33	33	33
WK6				21	14	64

F

PS	MV	WK1	WK2	WK3	WK5	WK6
MV	93	7				
WK1		73	13			
WK2		10	87	3		
WK3		3	7	66	7	17
WK5			12	36	28	24
WK6			17	21	8	54

G

RS	MV	WK1	WK2	WK3	WK5	WK6
MV	80	20				
WK1		63	23			
WK2	7	11	67	0		
WK3			4	22	44	30
WK5		5	11	21	47	16
WK6		7	7	33	13	40

Supplementary Figure S4. Ripening progress in GH, GS, and PS berry classes in relation to that of the RS berry class. A to C, Principle component analyses of the total soluble solid content, color parameters, and elasticity of individual GH (A), GS (B), or PS (C) berries (n = 30) at mid-*véraison* (V) and at one, two, or three weeks past-V compared to the corresponding data for RS berries (n = 30). Purple color denotes RS berries, and dark green, light green, and orange colors represent GH, GS, and PS berries, respectively. The stages V and 1, 2, and 3-weeks past-V are indicated with plus mark, closed circles, open circles, and closed squares, respectively. D to G, Discriminant analyses showing the ripening advancement of individual berries within a class. Numbers in the columns are the percentage of berries assigned to the corresponding times based on berry ripening parameters, Color Index, total soluble solid content, and Elasticity.
